# Heat Treatments for Minimization of Residual Stresses and Maximization of Tensile Strengths of Scalmalloy^®^ Processed via Directed Energy Deposition

**DOI:** 10.3390/ma17061333

**Published:** 2024-03-14

**Authors:** Rachel Boillat-Newport, Sriram Praneeth Isanaka, Jonathan Kelley, Frank Liou

**Affiliations:** Department of Mechanical Engineering, Missouri University of Science and Technology, Rolla, MO 65409, USA; rmb8t6@mst.edu (R.B.-N.);

**Keywords:** aluminum, additive manufacturing, directed energy deposition, laser-based process, Scalmalloy^®^, residual stresses, stress relief

## Abstract

Scalmalloy^®^ is an Al-Mg-Sc-Zr-based alloy specifically developed for additive manufacturing (AM). This alloy is designed for use with a direct aging treatment, as recommended by the manufacturer, rather than with a multistep treatment, as often seen in conventional manufacturing. Most work with Scalmalloy^®^ is conducted using powder bed rather than powder-fed processes. This investigation seeks to fill this knowledge gap and expand beyond single-step aging to promote an overall balanced AM-fabricated component. For this study, directed energy deposition (DED)-fabricated Scalmalloy^®^ components were subjected to low-temperature treatments to minimize residual stresses inherent in the material due to the layer-by-layer build process. X-ray diffraction (XRD) indicated the possibility of stress minimization while reducing the detriment to mechanical strength through lower temperature treatments. Microstructural analyses consisting of energy dispersion spectroscopy (EDS) and electron backscatter diffraction (EBSD) revealed the presence of grain growth detrimentally affecting the strength and elongation made possible by very small grains inherent to AM and rapid solidification. Tensile testing determined that treatment at 175 °C for 1 h provides the best relief from the existing residual stresses; however, this is accompanied by a diminishment in the yield and tensile strength of 19 and 9.5%, respectively. It is noted that treatment at 175 °C for 2 h did not provide as great of a decrease in residual stresses, theorized to be the result of grain growth and other strengthening mechanisms further stressing the structure; however, the residual stresses are still significantly diminished compared with the as-built condition. Furthermore, a minimal reduction of the tensile strengths indicates the possibility of finding a balance between property diminishment and stress state through the work proposed here.

## 1. Introduction

Aluminum alloys are commonly processed using a wide range of methods and employed in many applications where their favorable mechanical properties are most desired. Most structural parts are fabricated using traditional methods, such as casting, forging, extrusion, and powder metallurgy. Aluminum alloys are characterized by high strength-to-weight and stiffness-to-weight ratios [1], making them attractive for use in near-net-shape processes; however, these alloys also exhibit poor laser absorptivity due to high reflectivity, high thermal conductivity, and a strong tendency towards oxide formation [2,3]. These complications make aluminum alloys difficult to fabricate using AM processes.

In addition to the inherent properties of aluminum and its alloys, AM processing of these high-strength alloys is plagued by unique defect mechanisms. High-strength aluminum alloys, such as the commonly used Al7075 and Al6061, have a large mushy zone or solidification range [2,4]. When fabricated via AM, the large solidification range promotes cracking and poor mechanical behavior [4,5,6]. The tendency for cracking of many high-strength aluminum alloys and their high reflectivity to incident energy dramatically limits the number of viable alloys for use with AM, mostly to the casting aluminum alloy compositions; however, these alloys are not capable of achieving the high mechanical properties desired for many applications. Many aluminum alloys also fall prey to the vaporization of lower boiling elements [7]. This can not only contribute to the formation of porosity defects but can also impact the overall composition and, ultimately, the resulting mechanical performance.

Many studies have explored the use of heat treatments to improve the properties of AM components and, depending on the material, could include a series of steps [8,9]. After exploring the literature, it is clear that multistep and single-step post-processing treatments have a place for material enhancement. However, the consensus for many aluminum alloys, especially Scalmalloy^®^, is that direct aging is the best option, as additional treatments often negatively impact the fine microstructure achieved by the rapid solidification and degradation of the mechanical behaviors. As a result, many of the treatments commonly utilized in traditional processing, such as stress relief heat treatments, are not used. This becomes problematic as the rapid solidification and thermal gradients that characterize AM induce stresses in the material that remain after fabrication. Residual stresses can dramatically affect part dimensions, mechanical properties, and resistance to failure [10]. Kempen et al. noted the presence of cracking and delamination of powder bed-fabricated M2 tool steel parts as the result of high residual stresses [11]. Lu et al. determined that residual stresses induced significant warping of powder bed-fabricated Ti-6Al-4V thin-wall structures [12].

While residual stress minimization is clearly needed as a treatment step, minimization must be balanced such that mechanical behavior isn’t significantly diminished. Růžičková et al. explored the concept of intermediate temperature stress relief treatments to map the response of SLM-fabricated AlSi10Mg to different heat treatment factors [13]. The authors determined that heat treatment at 240 °C for 2/6 h followed by a furnace cool to 100 °C and air cooling resulted in a 15% decrease in tensile strength compared with the as-built components [13]. It was also noted that hold time at 240 °C cases did not significantly impact the tensile properties [13]. In contrast, treatment at 300 °C for 2 h followed by water quenching/air cooling resulted in greater degradation of the tensile strength [13]. Mfusi et al. explored the use of a 300 °C/2 h stress relief treatment on SLM AlSi10Mg and also noted a dramatic decrease in the tensile strengths (UTS: 420–470 MPa as-built/110–160 MPa after stress relief) and an improvement in the ductility [14]. Trevisan et al. also utilized a 300°/2 h stress relief treatment with SLM-fabricated A357 and noted a dramatic decrease in the Vicker’s microhardness, yield strength, and ultimate tensile strength [15]. The hardness decreased from 120 ± 2 HV0.1 to 81 ± 1 HV0.1 after stress relief [15]. The yield strength and tensile strength exhibited a reduction of 30 and 34%, respectively [15]. In contrast, the ductility was improved from 5.3 ± 0.4% to 8.3 ± 1.2% [15].

In this research, a theory has been developed in which the use of low/intermediate temperatures could lead to a reduction, if not elimination, of the residual stresses present in the components; however, the key is finding a treatment that finds a balance between residual stress minimization and the decrease in the mechanical behavior. It is worth noting that many stress-relieving treatments proposed in the literature have been noted to be not as effective until above 200 °C. Still, in finding the desired balance, total elimination of the residual stress is not mandatory. As such, temperatures lower than 200 °C may prove useful and allow for both minimization of the impact on mechanical properties while still eliminating a number of residual stresses. For this investigation, a series of low-temperature stress relief treatments were performed to evaluate the potential for residual stress relief coupled with minimal diminishment of tensile strength.

## 2. Materials and Methods

### 2.1. AM Processing

**Figure 1:**
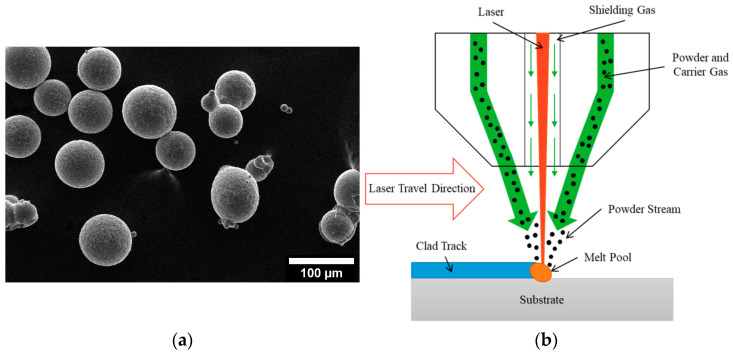
(**a**) Scalmalloy^®^ powder feedstock supplied by Toyal America, INC.; (**b**) Process schematic for DED. Reprinted with permission from Ref. [16], 2023, MDPI.

**Figure 2:**
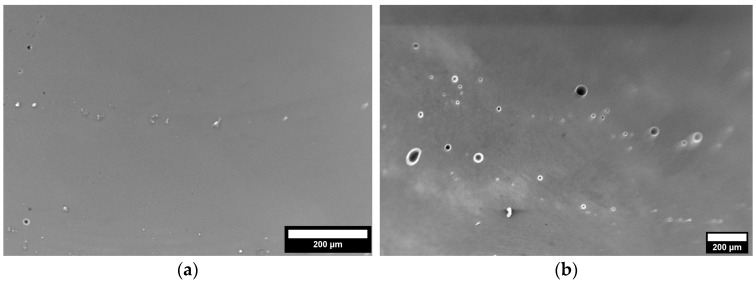
(**a**) Chosen parameter set showing minimal defects. (**b**) Representation of parameter sets resulting in high porosity.

**Figure 3:**
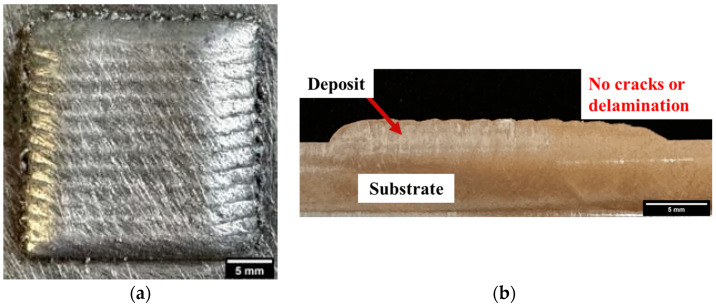
(**a**) Top view of 25 mm × 25 mm × 4 mm Scalmalloy^®^ coupon fabricated via DED; (**b**) side view of Scalmalloy^®^ coupon.

For this investigation, Scalmalloy^®^ powder was obtained from Toyal America, INC. (Lockport, IL, USA). Analysis of the particle morphology, shown in Figure 1a, shows good morphology with high sphericity and few to no satellites and agglomerates. Additively manufactured Scalmalloy^®^ components were fabricated using powder-fed directed energy deposition (DED), as shown in Figure 1b. The samples were deposited in an inert argon environment. Parameter optimization was performed using the selected parameters in Table 1. Optical microscopy was performed on each parameter case to determine the parameters yielding a final component with minimal microstructural defects. Figure 2a shows the relatively defect-free sample fabricated by the optimized parameters chosen for this study, which are given in Table 2. This parameter set minimized defect formation, making it the optimal parameter set for heat treatment investigations. A representative example of parameter sets that resulted in extensive defect formation is also shown in Figure 2b. Figure 3 gives a top and side view of a Scalmalloy^®^ DED coupon. From the side view, Figure 3b, it can be seen that there is good adhesion between the deposit and substrate, which is evidenced by the fact that there is no cracking or delamination. Archimedes density performed on these substrates matched the theoretical value from the manufacturer, indicating near fully dense parts.

### 2.2. Stress Relief Heat Treatments

A series of low-temperature stress relief treatments were chosen for implementation. The temperatures of interest are 150, 175, 200, 225, and 250 °C for hold times of 0.5, 1, and 2 h, followed by air cooling. It is worth noting that these treatments are considerably lower in temperature than the recrystallization temperature, which is increased considerably due to the formation of Al_3_Sc [17]. After post-processing, the samples underwent testing to determine the residual stresses present before and after each treatment. Air cooling was chosen as the final stage of treatment to minimize any potential grain growth that could occur when furnace cooling. Additionally, water quenching was not chosen, contrary to the results given by Růžičková et al., in which the decrease in tensile strength was noted to be greater when air cooling than water cooling [13]. This was chosen as the goal to minimize residual stresses. It was theorized that water quenching would induce greater stresses into the material due to the thermal shock inherent to this procedure.

### 2.3. Residual Stress Measurements

**Figure 4:**
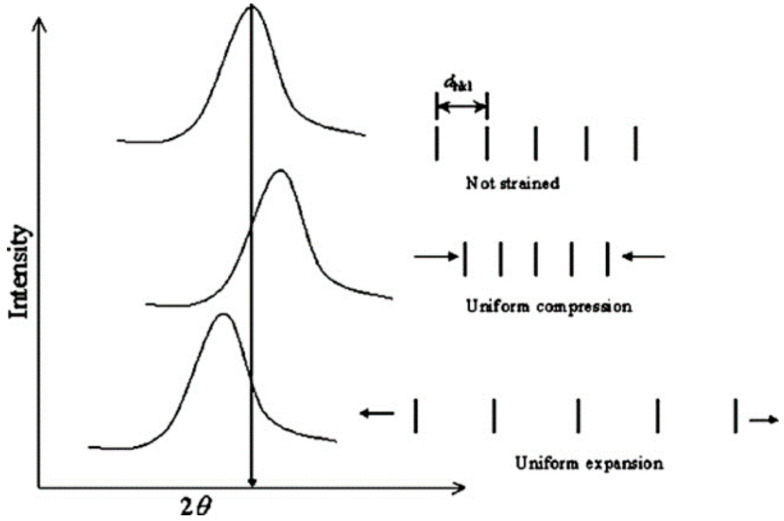
Impact of changing lattice spacing on diffraction patterns. Reprinted with permission from Ref. [18], 2005, Elsevier.

**Figure 5:**
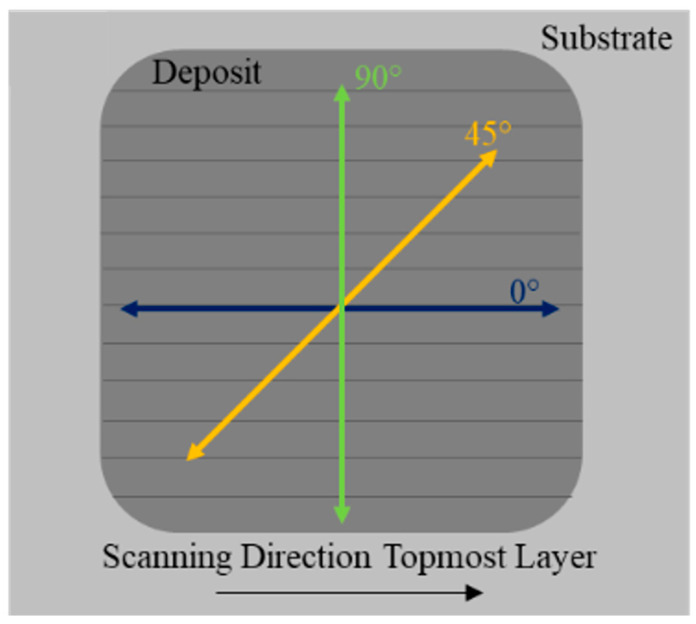
Orientation of directions analyzed during residual stress testing.

For testing residual stresses in the coupons, XRD was utilized to evaluate the sample coupons without destroying the samples. XRD uses surface residual stress analysis by collecting diffraction data along a specific orientation across a range of tilt angles away from the normal. It is worth noting that assuming a case of planar stress for the measured volume allows for the determination of an unstrained or starting lattice spacing, d, as approximately equivalent to the lattice spacing normal to the surface. A relationship exists between the diffraction pattern and the lattice spacing, such that when a material is under strain or changes strain state, the expansion or contraction of the material’s lattice structure changes the lattice spacing. These changes correlate to shifts in the diffraction pattern that can be measured to determine lattice spacing, as in Figure 4. Using the change in lattice spacing, it is possible to determine the strain and plot d vs. sin 2ψ, where ψ is the nominal tilt angle. The slope of the linear d vs. sin 2ψ distribution coupled with Young’s modulus and Poisson’s ratio allows for calculating the stresses present along the direction of measurement. Figure 5 shows the orientation of the residual stress measurements as it correlates with the track direction. Residual stress analysis was performed on the top surface of the deposit, examining local behavior.

### 2.4. Microstructural and Mechanical Characterization

Microscopy samples were prepared by polishing with SiC grit papers (400, 600, 800, and 1200 grit), followed by polishing with diamond slurry suspensions (6, 3, and 1 microns), and ending with a colloidal silica suspension (0.05 microns). Microstructural analysis was performed using a Thermo Fisher Scientific, Inc. (Waltham, MA, USA) PRISMA scanning electron microscope for electron microscopy imaging and energy-dispersive spectroscopy (EDS). XRD was performed using a Malvern Panalytical (Almelo, Netherlands) X’Pert Multipurpose Diffractometer with a copper X-ray source to determine the phase structures present. Electron backscatter diffraction (EBSD) was performed by JH Analytical using a Thermo Fisher Scientific (Brno, Czech Republic) Apreo S SEM with an Oxford Instruments (High Wycombe, Buckinghamshire, UK) Symmetry S2 EBSD with an accelerating voltage of 30 kV.

**Figure 6:**
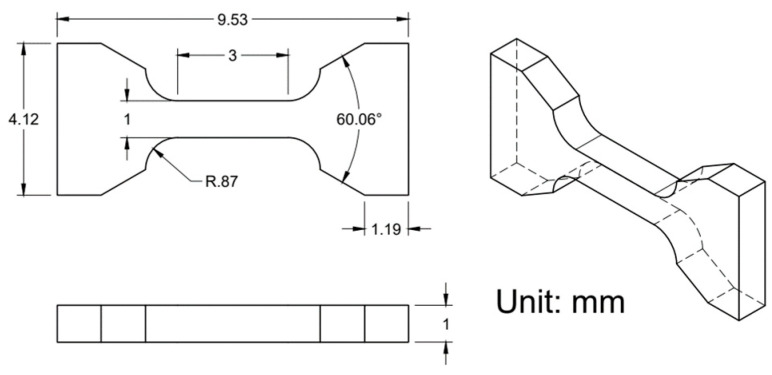
Drawing of miniature tensile specimens utilized in this study [19].

Tensile testing was performed using a Tinius Olsen Universal Testing Machine Model 25ST at a 0.25 mm/min positional rate. Miniature tensile specimens, shown in Figure 6, were cut out of the center of DED deposits in the XY plane using a Sodick Inc. VL600QH electrical discharge machine (EDM). Miniature samples were chosen for use in this study as they can be strategically located, and the interaction volumes are more sensitive to defects in AM materials, such as residual stresses. Before testing, all samples were hand polished to a metallurgical grit of 800 and measured to ensure all samples were within tolerance. Five tensile specimens were analyzed for statistically relevant results at each time and temperature combination. The corresponding dimensions were subsequently used to calculate the tensile strengths and strains in terms of median and ranges.

## 3. Results

### 3.1. Residual Stress Testing

**Figure 7:**
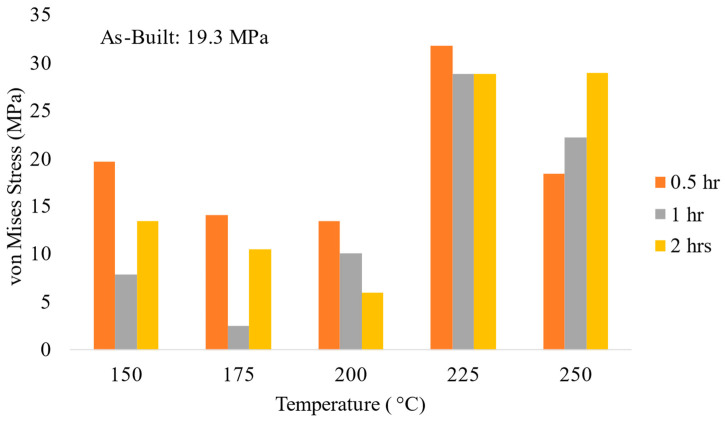
Calculated von Mises stresses displaying the overall change in stresses caused by heat treatments relative to the as-built case.

Figure 7 displays the von Mises stresses calculated using the XRD surface residual stress analysis results performed on the samples subjected to low-intermediate heat treatment relative to the untreated, as-built sample. The results correlate well with the elliptical fit used when calculating the residual stresses. All samples were fabricated under the optimized processing parameters; thus, any differences between the samples could be said to correspond to the heat treatments. Examination of the stresses present after 0.5 h for each temperature regime showed a minimal decrease in the residual stresses when heat treating for 0.5 h. The lowest stress was noted at 175 °C after 1 h of treatment. Increasing the time to 2 h, it is noted that the lowest stress value occurred at 200 °C; however, the stress noted is still higher than the stress at 175 °C and 1 h. Observation of the overall trends at each time regime shows a tendency towards a decrease in stress as temperature increases until around 225 °C when the stresses increase dramatically, exceeding the as-built case. 

While only the post-processing heat treatment should be the differing factor impacting the residual stresses, there is the possibility that the deposits themselves could be influencing the results. Between layers, a 90° rotation was applied to the raster pattern to minimize the residual stresses in the final samples. However, the penetration depth for the XRD residual stress analysis is estimated to be approximately 11 microns, while the overall layer thickness for the deposits was around 0.9 mm. This indicates that we are sampling only a portion of the topmost layer. While the penetration depth is not enough to sample more than a portion of one layer, it is hypothesized that the lower residual stress values seen with the as-built coupons could be a cumulative response to the chosen raster pattern and other factors during processing.

### 3.2. Tensile Properties

**Figure 8:**
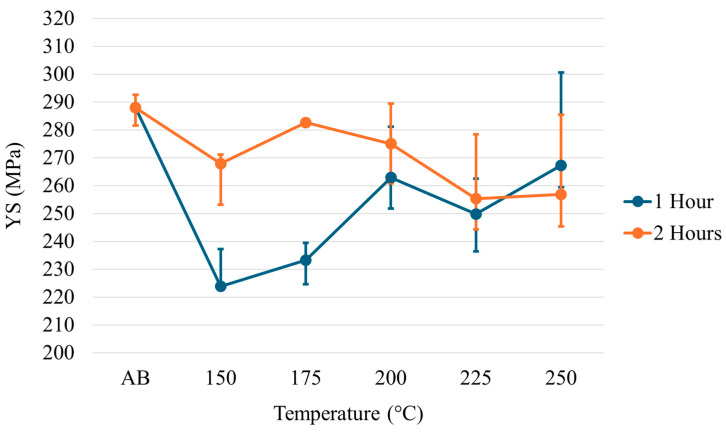
Yield strength of low-temperature heat-treated Scalmalloy^®^.

**Figure 9:**
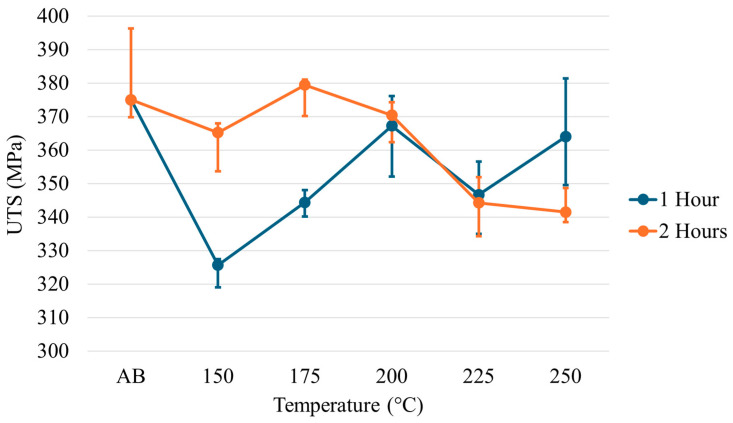
Ultimate tensile strength of low-temperature heat-treated Scalmalloy^®^.

**Figure 10:**
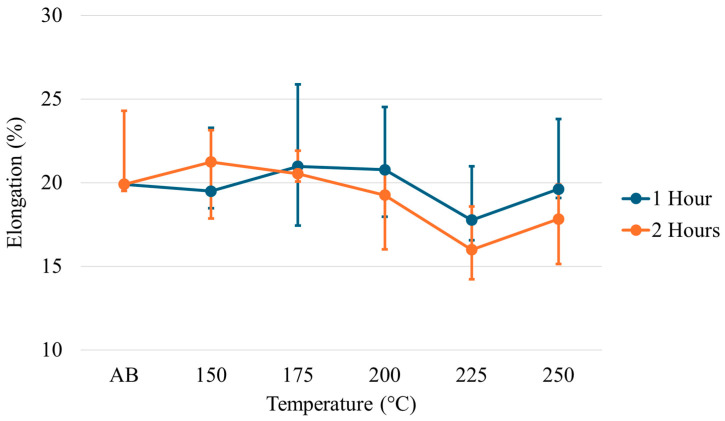
Elongation of low-temperature heat-treated Scalmalloy^®^.

The yield strength (YS), ultimate tensile strength (UTS), and elongation for each temperature across each time regime are given in Figure 8, Figure 9 and Figure 10. Examination of the YS and UTS results indicates that all heat treatments decreased strength when compared with the as-built condition. After 1 h, the YS drops dramatically for the heat treatments on the lower temperature end; however, as temperature increases, the YS increases to values just below the as-built condition. As the temperature increases at 2 h of treatment, a trend is seen in which the strength values decrease rather than increase, as seen at 1 h of treatment. Similar trends and features are seen for the ultimate tensile strength results. The elongation results show minimal changes relative to the as-built condition across all temperatures at the 1 h treatment. However, a slight decrease in elongation has been noted at higher temperatures after 2 h.

### 3.3. Microstructural Analysis

**Figure 11:**
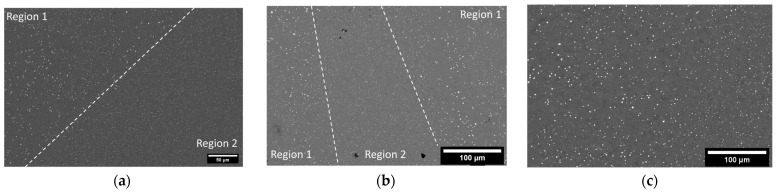
Microstructural evolution across increasing temperature: (**a**) as-built Scalmalloy^®^ deposit with the boundary marked between region 1 and region 2, (**b**) Scalmalloy^®^ deposit after heat treatment at 175 °C for 2 h with boundaries and regions 1 and 2 marked, and (**c**) Scalmalloy^®^ deposit after heat treatment at 250 °C for 2 h.

Microstructural features as an evolution of time are given in Figure 11. In the as-built and 175 °C/2 h conditions, regions are seen in which there exists a large volume of very bright precipitates and other regions where there are very few of these bright precipitates. Image analysis using ImageJ v1.53k was performed on SEM micrographs for the as-built, 175 °C at 2 h, and 250 °C at 2 h conditions. The images were thresholded such that only the precipitates were sampled. The thresholded images were then measured, and the maximum and minimum Feret’s diameters were determined; refer to Table 3. The area fraction and precipitate number were also found for Figure 11a–c to show that the increase in precipitate size with heat treatment correlates to a decrease in the number of precipitates.

**Figure 12:**
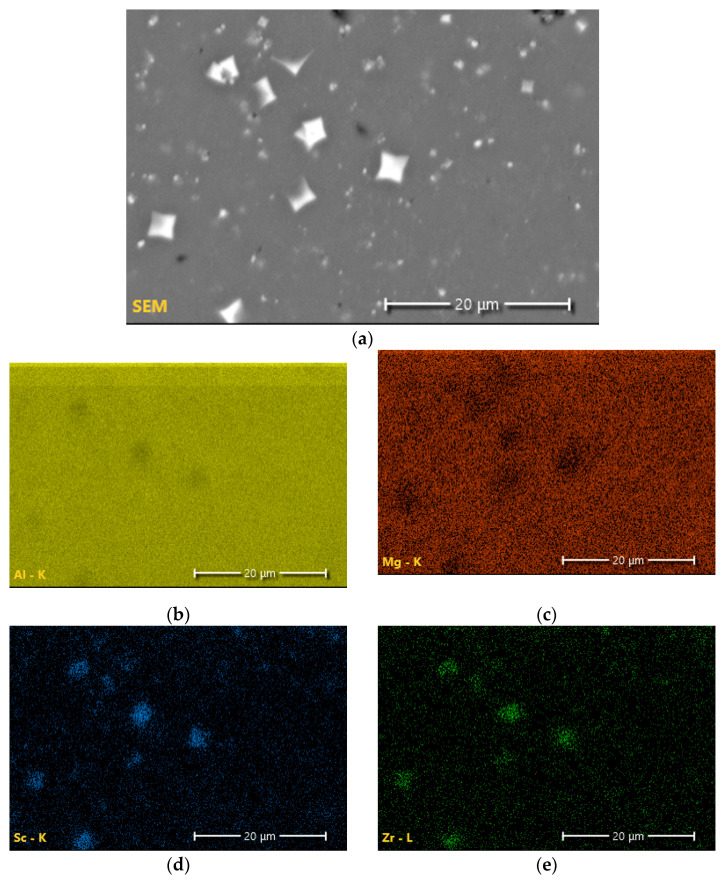
Energy-dispersive spectroscopy elemental maps depicting the elemental distribution of the matrix region and the Al-Mg-Sc-Zr precipitate phase: (**a**) SEM image of the region of interest, (**b**) element map of aluminum, (**c**) elemental map of magnesium, (**d**) elemental map of scandium, and (**e**) elemental map of zirconium.

From literature and using EDS, as shown in Figure 12, these very bright precipitates in Region 1 are Al-Sc-Zr precipitates believed to be Al_3_(Sc,Zr). Of note is the presence of an additional phase in Region 2, which was determined to be Al-Mg in composition, with 4 wt% Mg. XRD phase results for both as-built and heat-treated conditions revealed the presence of only aluminum phase regardless of temperature and time. The results from SEM and EDS in Figure 12 directly contradict this.

**Figure 13:**
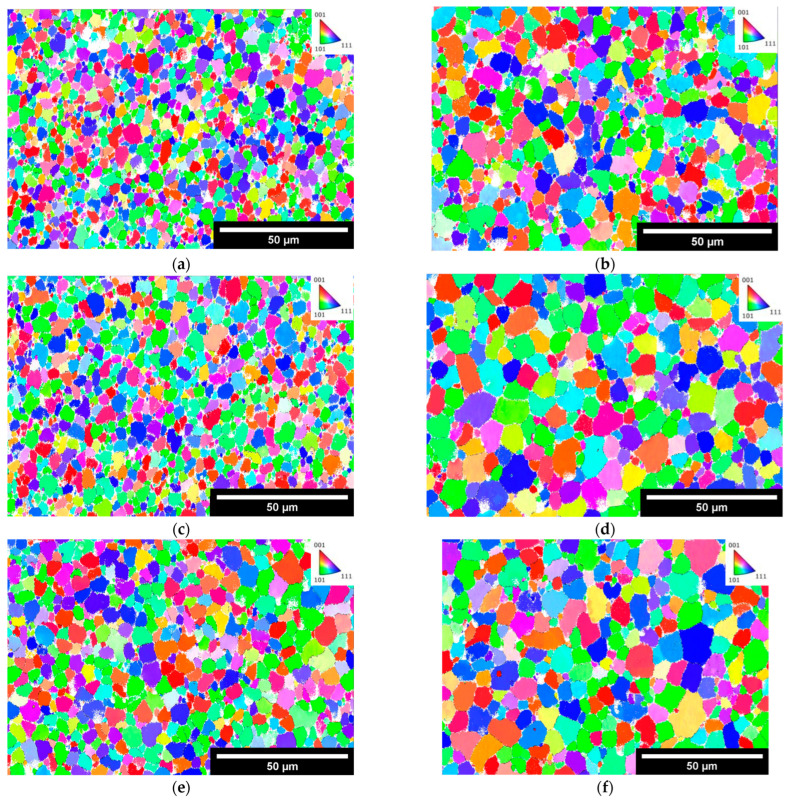
EBSD results showing grain orientation and providing grain size data for as-built (**a**,**c**,**e**) and HT 250 °C for 2 h (**b**,**d**,**f**).

Figure 13 shows the results of electron backscatter diffraction (EBSD) of the Scalmalloy^®^ deposits in the as-built and 250 °C for 2 h in a heat-treated condition. Examination of the microstructure before and after heat treatment shows a distinct increase in grain size. This is further supported by the grain size analysis of the results, given in Table 4.

## 4. Discussion

### 4.1. Residual Stresses

**Figure 14:**
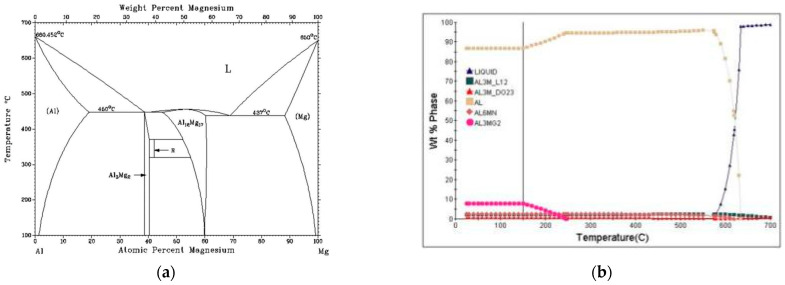
(**a**) Al-Mg binary phase diagram. Reprinted with permission from Ref. [20]; 2015, Taylor & Francis. (**b**) JMatPro phase evolution for Scalmalloy^®^ during isothermal heat treatment.

Considering the relationship between the residual stresses and tensile strengths, it is clear that residual stresses cannot be minimized without a corresponding decrease in tensile strength. The greatest residual stress minimization occurred at 175 °C after 1 h of treatment. In response, the YS and UTS decreased by 19.0% and 9.5%, respectively. While stresses were minimized at lower temperatures and longer times, residual stresses at higher temperatures (225 and 250 °C) did not exhibit diminishment. In fact, the residual stresses were greater than those found in the as-built condition. On examination of the microstructure at lower temperatures (refer to Figure 11), it is clear that there is an Al-Mg phase present that is not seen at higher temperatures. From XRD analysis, the only phase found was aluminum; however, examination of the literature and the microstructure indicate that it is possible for phases that are both small and have a low content in the alloy to not be found by XRD but can still exist. Standardized EDS determined that the phase was primarily aluminum, with approximately 4 wt% magnesium. Upon examination of a binary Al-Mg phase diagram at 4 wt% magnesium, at temperatures around 235 °C, there is an Al_3_Mg_2_ phase in which the magnesium dissolves into the matrix with increasing temperatures (Figure 14a). This theory is further backed using JMatPro v14 for the composition of Scalmalloy^®^ to explore the impact of increasing temperatures on phase development (Figure 14b). The impact on residual stresses caused by phase changes and formation can result in changes in the volume, resulting in expansion or contraction of the metal. Thus, residual stresses can be invoked, as seen in the higher temperature regimes of this study.

### 4.2. Heat Treatments and the Impact on Tensile Strength

The amount of tensile strength diminishment is directly related to the strengthening mechanisms at play and the impact that the heat treatment (required for stress relief) has on these mechanisms. From the literature, three main strengthening mechanisms have been noted for Scalmalloy^®^: (1) Hall–Petch strengthening, (2) solid solution strengthening, and (3) precipitation strengthening [21,22]. It is worth noting that other potential strengthening mechanisms could be active; however, this work will only cover the most dominant. The strength of a material, especially metallic materials, is influenced by the motion of dislocations, and by hindering these dislocations, it is possible to strengthen a material.

Hall–Petch or grain boundary strengthening relies on the size of the grains to hinder dislocation motion [23,24]. Grain boundaries are essentially barriers that hinder dislocation motion, and when grain size is smaller, there are more barriers to hinder motion [23,24]. Additive manufacturing has the distinct advantage of rapid cooling rates, which promote very fine grains in the as-built state. As a result, many post-processing heat treatments are single-stage steps to minimize grain growth while allowing for precipitation, as a significant amount of Scalmalloy^®^’s strengthening results from the fine grain size.

Solid solution strengthening results from additional atoms not part of the base material distorting the lattice and generating strains [23,24]. These strains act as a barrier to dislocation motion and strengthen the material [23,24]. Solid solution strengthening plays a role in AM due to the rapid cooling rates, allowing little time for precipitation. Scalmalloy^®^ exhibits solid solution strengthening in the as-built case, as much of the alloying elements remain in solid solution, with only a small amount being precipitated in the form of nucleating phases.

Precipitation strengthening occurs due to the presence of hard precipitates throughout the microstructure, which work to pin dislocation motion [23]. A dislocation must either bypass the precipitate or Orowan looping or shear it to continue moving [23]. Strong precipitates do not allow for shearing [23]. Orowan looping results from dislocations bowing during the attempt to bypass a precipitate [23]. The act of bowing can result in dislocation loops around the precipitates [23]. These loops decrease the space available for additional dislocation motion [23]. Successful precipitation strengthening occurs when bypassing and shearing a precipitate cannot occur, causing the dislocation to be impeded [23].

In assessing the above cases for the Scalmalloy^®^ under investigation here, precipitation strengthening plays a role in providing strength. As the time and temperature increased with each case, the size of Al-Sc-Zr precipitates increased from the as-built case to the 250 °C/2 h HT case. With the increase in size, it was also noted that the number of precipitates decreased, which is theorized to be linked to the larger precipitates growing at the expense of the smaller precipitates. Considering Figure 11, the number of precipitates of the as-built (Figure 11a), HT 175 °C/2 h (Figure 11b), and HT 250 °C/2 h (Figure 11c) are 5912, 5402, and 1766, respectively. It is clear that the number of precipitates decreases with higher treatment temperatures and is accompanied by an increase in max Feret’s diameter from 0.693 ± 0.591 μm in the as-built case, 0.922 ± 0.688 μm in the 175 °C/2 h case, and 0.940 ± 0.810 μm in the 250 °C/2 h case. This severely limits the capability of precipitation strengthening, which could be a contributing factor to the diminishment in strength. Additionally, as with the increase in precipitate sizes, an increase in overall grain size was noted in the heat-treated samples (3.55 ± 2.54 μm, 5.05 ± 3.17 μm, 4.90 ± 3.39 μm) relative to the as-built samples (2.83 ± 1.57 μm, 3.02 ± 1.75 μm, 3.97 ± 2.19 μm). A considerable amount of strengthening for AM components comes from the very small grains in the as-built condition, which then shifts to precipitation strengthening after heat treatment as grain growth occurs during heat treatment.

Of note, when considering the tensile properties at 1 h treatment time, the strength at 200 °C and below is lower than the strength at 225 and 250 °C. As stated above, it is theorized that the Al-Mg phase is going back into solution, which can create additional stress in the material, increasing residual stress. The dissolution of this phase can further promote solid solution strengthening, which could offset any negative effects and increase strength. However, after 2 h, the YS and UTS at 225 and 250 °C decrease dramatically compared to the as-built measurements. This is theorized to be directly related to the currently active strengthening mechanisms being overshadowed by the growth of grains caused by longer treatment times at higher temperatures. It is worth noting that while the amount of growth seems small, this analysis was performed in the XY plane and thus does not include the large textural columnar grains that characterize the Z direction.

## 5. Conclusions

The use of low-temperature heat treatments to minimize residual stresses while maximizing the tensile properties of Scalmalloy^®^ fabricated using directed energy deposition has been explored. From the results, the following conclusions are drawn:

1. A decrease in tensile strength accompanies successful stress relief. The greatest stress minimization occurred at 175 °C after 1 h of treatment; however, the YS and UTS diminished by 19 and 9.5%, respectively. Treatment at 175 °C for 2 h exhibited less stress minimization than treatment at 175 °C for 1 h; however, when compared to the as-built condition, minimization of residual stresses is present at a considerable 45.6% decrease. Furthermore, with the decrease in residual stresses, the samples exhibited very minimal diminishments in average yield and tensile strength of 1.75% and 0.92%, respectively. The second-greatest minimization of residual stresses occurred at 200 °C after 2 h. The residual stresses were decreased by 68.9% compared with the as-built condition. This treatment also showed minimal diminishments in average yield and tensile strength of 5.09% and 2.99%, respectively. The results show the possibility for balance between stress minimization and property diminishment.
2. As-built AM components exhibit very fine grains due to the rapid solidification process. These fine grains provide considerable strengthening to the material. Subsequent heat treatment causes these grains to grow, reducing the effectiveness if not eliminating the benefits of Hall–Petch strengthening.
3. At heat-treatment temperatures of 225 and 250 °C, the residual stresses were increased and even exceeded the stresses in the as-built condition. This is theorized to be the result of magnesium in the phase Al_3_Mg_2_ dissolving into solution.
4. Observation of the microstructure found that grain coarsening was present with increasing temperatures and times despite the low treatment temperatures. This negatively impacts the potential for Hall–Petch strengthening and further contributes to the diminishment of tensile strength.

Exploration into the field of stress relief for AM-fabricated components is of considerable benefit, as these residual stresses have the potential to lead to part failure and dimensional warpage and negatively impact mechanical behavior. However, quantifying and eliminating these stresses is not straightforward due to the fabrication method’s complexity. AM processing involves several parameters, such as laser power and scanning speed. Additionally, the raster patterns applied both in layers and across layers complicate matters. Finally, AM is not a single method of fabrication. There are several different AM processes, each with unique incident energy characteristics, thermal profiles, and solidification behaviors. As a result, it is difficult to determine the directions in which stresses can be acting, which limits the methods that can be used to evaluate the stresses.

The benefit of this work stems from the concept of multistage heat treatments that address multiple properties of the material rather than just a single-stage treatment, as seen with many AM aluminum compositions. While effective for maximizing tensile strength, these single treatments fail to address the substantial issue of residual stresses caused by the layer-by-layer manufacturing process. This work is the first step in building the much-needed multistep heat treatments tailored for AM while still providing the ability to address more than one property.

## Figures and Tables

**Table 1:** Design of experiments for optimization of deposition parameters.

ID	Power (W)	Scan Speed (mm/s)	Feed Rate (g/min)
1	1500	12.5	2.25
2	1750	12.5	2.25
3	1500	17.5	2.25
4	1750	17.5	2.25
5	1500	12.5	3.25
6	1750	12.5	3.25
7	1500	17.5	3.25
8	1750	17.5	3.25
9	1500	15.0	2.75
10	1750	15.0	2.75
11	1625	12.5	2.75
12	1625	17.5	2.75
13	1625	15.0	2.25
14	1625	15.0	3.25
15	1625	15.0	2.75

**Table 2:** Optimized deposition parameters.

Parameters	
Power (W)	1625
Scan Speed (mm/s)	15
Powder Feed Rate (g/min)	2.25

**Table 3:** Maximum and minimum ferret diameter of precipitates present in as-built Scalmalloy^®^, HT Scalmalloy^®^ at 175 °C for 2 h, and HT Scalmalloy^®^ at 250 °C for 2 h. Area fraction and number of precipitates corresponding to the micrographs in Figure 11.

Sample	Avg Max Feret Diameter	Avg Min Feret Diameter	Area Fraction Figure 11	Number of Precipitates Figure 11
As-Built	0.693 ± 0.591	0.443 ± 0.393	3.85%	5912
175 °C/2 h	0.922 ± 0.688	0.596 ± 0.456	2.95%	5402
250 °C/2 h	0.940 ± 0.810	0.622 ± 0.564	1.79%	1766

**Table 4:** Grain size results for as-built Scalmalloy^®^ and HT Scalmalloy^®^ at 250 °C for 2 h using equivalent circle diameter.

Sample	Figure 13 ID	Avg Grain Size (μm)	Max Grain Size (μm)
As-Built	A	2.83 ± 1.57	8.23
	C	3.02 ± 1.75	9.22
	E	3.97 ± 2.19	11.21
250 °C 2 h	B	3.55 ± 2.54	12.82
	D	5.05 ± 3.17	14.86
	F	4.90 ± 3.39	16.48

## Data Availability

Data are contained within the article.
